# Schmidt’s syndrome presenting as a generalised anxiety disorder: a case report

**Published:** 2013-12-25

**Authors:** D Anyfantakis, EK Symvoulakis, I Vourliotaki, S Kastanakis

**Affiliations:** * Primary Health Care Centre of Kissamos, Crete, Greece; ** Private Family Practice Unit in Heraklion, Crete, Greece; *** Department of Endocrinology and Metabolism, Venizeleio General Hospital of Heraklion, Crete, Greece; **** First Department of Internal Medicine, Saint George General Hospital of Chania, Crete, Greece

**Keywords:** Schmidt’s syndrome, autoimmune thyroiditis, autoimmune polyglandurar syndrome type 2, generalized anxiety disorder

## Abstract

Abstract

Schmidt’s syndrome or autoimmune polyglandurar syndrome type 2 represents an uncommon endocrine disorder composed by Addison’s disease with autoimmune thyroid disease and/or type 1 diabetes mellitus. The syndrome usually affects women in the fourth decade of their lives. Prompt diagnosis and treatment can prevent serious complications.

We present the case of a 64-year-old woman with generalised anxiety, facing socio-economic problems. Her symptoms attributed to stress led to a late diagnosis. Physicians involved have to be aware about endocrine disorders of which first manifestations may have atypical components mimicking mental health problems.

## Introduction

Schmidt’s syndrome also known as autoimmune polyglandular syndrome type 2 (APS type 2) is a rare endocrine disorder defined by the combined occurrence of primary adrenal insufficiency with autoimmune thyroid disease and/or type 1 autoimmune diabetes [**1**]. The condition was first described by Schmidt in 1926 reporting two patients with Addison’s disease and chronic lymphocytic thyroiditis and was subsequently named after him [**2**]. The rarity of the condition and the atypical presentation of adrenal insufficiency and hypothyroidism often lead to misdiagnosis with life-threatening consequences for the patient [**1**]. We report a case of Schmidt’s syndrome presenting with an anxiety disorder. 

## Case Report

A 64-year-old Caucasian female, attended a rural primary care setting complaining of difficulty concentrating, insomnia and intermittent fatigue, progressively worsening over the last six months. She attributed her state to an excessive, uncontrollable almost daily, worry about the financial status of her family caused by the economical crisis. Her symptoms compromised her occupational and social functioning. Personal background was negative for depression, social phobia, obsessive-compulsive disorder or post-traumatic stress disorder.

The patient’s prior medical history was unremarkable except for a Hashimoto’s thyroiditis, treated with sodium levothyroxine 100μg daily. The diagnosis was made 5 years ago and was based on thyroid ultrasonographic features, raised thyroid stimulating hormone as well as anti-thyroid antibodies (anti-antithyroid peroxidase and anti-antithyroglobulin antibodies). Current physical examination did not yield any relevant findings. From the laboratory work-up, complete blood count, folate and vitamin B12 levels were normal. Thyroid function tests were within normal limits except for the presence of anti-thyroid antibodies.

The patient was referred for psychiatric evaluation. Based on the Diagnostic and Statistic Manual of Mental Disorders, Fourth Edition (DSM-IV), the patient was diagnosed by the psychiatrist with generalized anxiety disorder [**3**] and (was) prescribed benzodiazepine and an antidepressant agent (bromazepam 1.5 mg p.o and escitalopram 10 mg p.o daily).

Within a month after pharmacological treatment, the patient was readmitted to the local primary health carer center due to the worsening of asthenia that required bed rest, and mild gastrointestinal distress associated with altered bowel habits. Skin examination revealed dehydration and a previously absent diffuse hyperpigmentation of extensor surfaces (elbows and knees). She also presented with orthostatic hypotension with blood pressure levels on the supine and sitting position 90/70 mmHg and 70/57mmHg respectively. The rest of her vital signs were as it follows: body temperature, 37o C; heart rate of 115 beats/min; oxygen saturation 97% while she was breathing ambient air. Cardiac and abdominal examination was unremarkable and electrocardiogram revealed sinus tachycardia. The previous diagnosis of thyroiditis combined with the current clinical condition raised the suspicion of an autoimmune endocrine syndrome. A transfer to a secondary care center was immediately arranged.

**Fig. 1:**
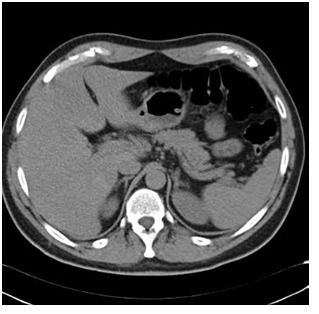
Computed tomography imaging of the adrenal glands

Current laboratory investigations included a total blood count, blood biochemistry tests and thyroid function tests which showed: glucose, 3.89 mmol/L; serum sodium, 134 mmol/L; urea, 19.6 mmol/L; creatinine, 0.08 mmol/L; serum potassium, 5 mmol/L; alanine transaminase, 33 IU/mL; aspartate transaminase, 29 IU/mL. Thyroid function tests were normal except for the elevated levels of serum antithyroglobulin (394.7 IU/mL; normal range: 5 IU/mL to 60 U/ml) and antithyroid peroxidase (447.3 IU/mL; normal range: 10 IU/mL to 115 IU/ml). Adrenal function tests showed elevated plasma adrenocotropic hormone (ACTH) (1050 pg/ml)(231 pmol/l) and low fasting (7:30 am) plasma cortisol levels 0.055 μmol/L reaching to 0.11 μmol/L 60 min after injecting 250 μg IV cosyntropin (synthetic ACTH). Computed tomography (CT) imaging showed morphologically normal adrenal glands (Fig. 1). Detection of circulating adrenal cortex autoantibodies confirmed the diagnosis of Schmidt’s syndrome.

Initial fluid resuscitation included IV administration of 0.9% physiological saline solution with rate 1 liter/h. Hydrocortisone administration was initiated, 10 mg po in the morning, 5mg po at lunchtime and 5 mg in the early evening. Furthermore, 0.1 mg fludrocortisone was given. On discharge, the patient’s physical status was significantly improved and her anxiety remised within 1 week. During a 12-month follow up the patient’s psychological status was normal without recurrence of anxiety symptoms.

## Discussion

Schmidt’s syndrome is an uncommon disorder with an estimated prevalence of 1.4-2 per 100.000 [**4**]. It occurs most frequently between 30 and 40 years of age with predominance in female gender [**1**]. With regard to genetics, a strong association was reported between class II human leukocyte antigen haplotypes DR3, HLA DR4 and component disorders of the syndrome [**1**]. In terms of the sequence of the development of endocrine gland insufficiency in the Schmidt’s syndrome, it has been reported that in one half of the cases, autoimmune adrenal insufficiency is the abnormality that occurs first [**4**] while Hashimoto’s thyroiditis tends to occur simultaneously or after the emergence of autoimmune Addison’s disease [**5**]. Adrenal cortex antibodies or 21-Hydroxilase antibodies are detectable in the majority of the patients [**1**,**4**]. Treatment is based on the hormonal replacement of the component endocrinopathies [**1**].

In 1855, Thomas Addison, an English physician first described a disorder characterised by hyperpigmentation and salt wasting [**6**]. Addison’s disease represents a potential fatal condition that results from cortisol and aldosterone deficiency. It is also a rare clinical entity with an estimated prevalence of 117 per million in Europe [**7**]. In the vast majority of cases, it is caused by a non-specific autoimmune destruction of the adrenal cortex [**8**]. Other causes include infectious (tuberculosis), neoplastic (primary, metastatic), iatrogenic (surgery, medication) or vascular (haemorrhage, emboli) procedures [**8**]. 

Clinical manifestations of Addison’s disease at onset are often atypical. Fatigue, anorexia, dizziness, muscle and joint pain, decreased libido may be some of the clinical features at the early stages of the disease [**8**]. Psychiatric manifestations are also well-documented [**9**]. Publishing data in the 1940s reported that almost 7 out of 10 patients with the Addison’s disease presented with psychiatric symptoms [**9**]. Among these, depression was the most frequent while psychosis, catatonia and self-mutilation were the most unusual [**9**]. Remarkably, depression has been repeatedly described as the first symptom leading patients with Addison’s disease to seek medical care [**10**-**12**]. Although well established, the neuropsychiatric profile of Addison’s disease has received limited attention in the recent literature [**9**]. Glucocorticoid deficiency, electrophysiological, electrolyte and metabolic abnormalities are some of the probable causative mechanisms for the psychiatric manifestations associated with the disease [**9**]. 

The non-specific initial presentation makes the early diagnosis of adrenocortical insufficiency a difficult issue. For this reason, Addison’s disease is often overlooked and misdiagnosed by the involved physicians [**13**]. Furthermore, even psychiatrists seem to have limited awareness about the psychiatric manifestations on the early stages of the disorder [**9**]. 

Similarly, although Schmidt’s syndrome is more prevalent compared to the other combinations of APS type 2, initial symptoms may mimic a variety of other conditions making its diagnosis challenging [**1**]. Therefore, in our case, the atypical initial presentation with generalised anxiety assessed with terms of the patient’s financial and social distress resulted to a late diagnosis. Furthermore, the sequence of the autoimmune gland insufficiency was unusual. Although 1 out of 2 of the patients with autoimmune adrenal insufficiency may develop autoimmune thyroiditis, only 1 out of 100 of the patients with thyroid disease will develop adrenal insufficiency [**1**]. 

## Conclusions

It is remarkable that 1 out of 4 of the patients suffering from one autoimmune disease will develop another during their lifetime [**5**]. For this reason, physicians have to be aware of the clustering tendency of autoimmune diseases within the same individual. In this direction, a high level of diagnostic suspicion for Schmidt’s syndrome is required, especially when they encounter patients with a monoglandular autoimmune disease and atypical somatic or psychological complaints. Furthermore, screening of organ specific autoantibodies in these patients could help in detection of those at risk of developing Schmidt’s syndrome [**14**]. This is also important because initiation of thyroid hormone in a patient with autoimmune thyroiditis and misdiagnosed adrenal insufficiency may precipitate an acute adrenal crisis [**1**]. This happens because thyroxine stimulates increased hepatic metabolism of corticosteroids [**14**]. It is also remarkable that thyroid disorders may manifest with a wide variety of psychological symptoms [**15**]. Therefore, the physician’s familiarity with the non-specific clinical manifestations of the endocrine autoimmune disorders may allow for correct diagnosis and management at the early subclinical stages reducing negative outcomes.

**Consent**

 Written informed consent was obtained from the patient for publication of this case report and accompanying images. A copy of the written consent is available for review by the Editor-in-Chief of this journal.

**Conflict of Interests**

The authors declare that they have no competing interests
